# Genetic inactivation of mitochondria-targeted redox enzyme p66ShcA preserves neuronal viability and mitochondrial integrity in response to oxidative challenges

**DOI:** 10.3389/fphys.2012.00285

**Published:** 2012-07-20

**Authors:** Kimmy Su, Dennis Bourdette, Michael Forte

**Affiliations:** ^1^Vollum Institute, Oregon Health and Science UniversityPortland, OR, USA; ^2^Department of Neurology, Oregon Health and Science UniversityPortland, OR, USA; ^3^VA MS Center of Excellence-West and Neurology Service, Department of Veterans AffairsPortland, OR, USA

**Keywords:** oxidative stress, p66ShcA, mitochondria, neuronal viability

## Abstract

Mitochondria are essential to neuronal viability and function due to their roles in ATP production, intracellular calcium regulation, and activation of apoptotic pathways. Accordingly, mitochondrial dysfunction has been indicated in a wide variety of neurodegenerative diseases, including Alzheimer's disease (AD), Huntington's disease, amyotrophic lateral sclerosis, stroke, and multiple sclerosis (MS). Recent evidence points to the permeability transition pore (PTP) as a key player in mitochondrial dysfunction in these diseases, in which pathologic opening leads to mitochondrial swelling, rupture, release of cytochrome c, and neuronal death. Reactive oxygen species (ROS), which are inducers of PTP opening, have been prominently implicated in the progression of many of these neurodegenerative diseases. In this context, inactivation of a mitochondria-targeted redox enzyme p66ShcA (p66) has been recently shown to prevent the neuronal cell death leading to axonal severing in the murine model of MS, experimental autoimmune encephalomyelitis (EAE). To further characterize the response of neurons lacking p66, we assessed their reaction to treatment with stressors implicated in neurodegenerative pathways. Specifically, p66-knockout (p66-KO) and wild-type (WT) neurons were treated with hydrogen peroxide (H_2_O_2_) and nitric oxide (NO), and assessed for cell viability and changes in mitochondrial properties, including morphology and ROS production. The results showed that p66-KO neurons had greater survival following treatment with each stressor and generated less ROS when compared to WT neurons. Correspondingly, mitochondria in p66-KO neurons showed diminished morphological changes in response to these challenges. Overall, these findings highlight the importance of developing mitochondria-targeted therapeutics for neurodegenerative disorders, and emphasize p66, mitochondrial ROS, and the PTP as key targets for maintaining mitochondrial and neuronal integrity.

## Introduction

The importance of mitochondrial function in the integrity and stability of neurons and neuronal networks is well established (Hajnóczky and Hoek, 2007; Rizzuto et al., 2008; Szabadkai and Duchen, 2008; Duchen and Szabadkai, 2010). In addition to ATP synthesis to maintain neuronal ion gradients, axonal transport, and all synthetic functions, mitochondria also represent a repository of prominent regulators of neuronal apoptosis and an important neuronal Ca^2+^ store. Not surprisingly then, given their involvement in key neuronal functions, persistent mitochondrial dysfunction has been hypothesized to be important to the pathogenesis of common neurological disorders including neurodegenerative conditions such as Alzheimer's disease (AD), Parkinson's disease (PD), amyotrophic lateral sclerosis (ALS), and multiple sclerosis (MS) (Lu et al., 2000; Mattiazzi et al., 2002; Damiano et al., 2006; Dutta et al., 2006; Rui et al., 2006; De Vos et al., 2007; Sasaki and Iwata, 2007; Bueler, 2009; Wang et al., 2009; Narendra et al., 2010; Kim-Han et al., 2011).

A key mediator of mitochondrial function and dysfunction in neurons is the permeability transition pore (PTP), which can pathologically open via inducers such as Ca^2+^ and reactive oxygen species (ROS) leading to neuronal death (Bernardi et al., 2006). Consequently, modification of the pore, in particular through pharmacological or genetic manipulation of a regulatory component cyclophilin D (CyPD), has been shown to provide axonal protection in murine models of multiple neurodegenerative diseases, including AD, PD, ALS, MS, and stroke (Schinzel et al., 2005; Forte et al., 2007; Du et al., 2008; Martin et al., 2009; Wang et al., 2009).

PTP transitions between open and closed states can be regulated at many levels; consequently, misregulation of these upstream pathways may lead to persistent, pathological activation of the PTP. It has also become clear that ROS are potent inducers of the PTP through oxidative mechanisms that can function under both physiological and pathological conditions (Petronilli et al., 1994; Danial and Korsmeyer, 2004). An intriguing example of this mode of regulation has recently been ascribed to p66ShcA (p66), a specific splice variant of the ShcA gene (Ravichandran, 2001). In contrast to other ShcA isoforms (p52ShcA and p46ShcA), p66 is not involved in Ras regulation; rather, p66 contains an atypical mitochondrial targeting sequence (Migliaccio et al., 1997). Under normal conditions, 40% of p66 is localized to mitochondria, where it can act as an oxidoreductase by accepting electrons from reduced cytochrome *c*. Subsequently, these electrons are used to reduce molecular oxygen in the generation of O_2_^−^ and then hydrogen peroxide (H_2_O_2_), driving PTP opening (Giorgio et al., 2005; Pinton et al., 2007; Pellegrini and Baldari, 2009). Indeed, a variety of studies have supported a model in which a “stress sensing complex” keeps p66 inactive as long as stress levels remain moderate (Gertz et al., 2009; Gertz and Steegborn, 2010). However, under conditions of increased cellular stress, p66 has been proposed to function in a positive feed-forward loop, whereby ROS stresses lead to increased levels of p66-generated ROS, which ultimately induce cell death through persistent PTP opening. In such a model, the PTP has been proposed to constitute the immediate downstream target of mitochondrial p66 action in the activation of cell death pathways, a hypothesis that is in keeping with the recent demonstration that O_2_^−^ sparks may be one of the key triggers for PTP opening *in situ* (Wang et al., 2008).

Consistent with this model, our previous work has demonstrated a key role for p66-generated stresses in the propagation of neurodegenerative disease; genetic inactivation of p66 reduced the extent of axonal damage in spinal cords and optic nerves following EAE induction (Su et al., 2012) as did elimination of CyPD (Forte et al., 2007), establishing a functional interaction between p66 and the PTP on an *in vivo* level. To further our understanding of how p66 elimination promotes neuroprotection, here we have compared hippocampal neurons lacking p66 with control, wild-type (WT) neurons in the presence of challenges implicated in the axonal degeneration responsible for permanent disability in MS. Specifically, to assess responses to ROS, neurons were assessed by following treatment of cultures with H_2_O_2_ and to reactive nitrogen species (RNS) following treatment with DETA-NO. In addition to assessing viability, we have examined the morphology of mitochondria in response to these stresses, as previous studies have shown that morphology changes correlate with eventual neuronal damage under various noxious conditions associated with neurodegenerative diseases (Solenski et al., 2002; Nikic et al., 2011). Mitochondria, which are normally thin and elongated in morphology, become swollen and fragmented prior to notable structural damage of axons, and therefore may serve as a key marker of neuronal distress. Furthermore, increased intracellular production of oxidative agents, in particular, mitochondria-localized ROS, has been shown to exacerbate neuronal distress in multiple neurodegenerative disease models (Fiskum et al., 2003; Scherz-Shouval and Elazar, 2007). In this report, we demonstrate that neurons lacking p66 exhibit protection in response to oxidative challenges as well as preservation of mitochondrial morphology and reduction of mitochondrial ROS production. These results strengthen the theory that mitochondria-targeted redox enzyme p66 functions as a direct upstream activator of PTP-mediated neuronal death in neurodegenerative diseases.

## Materials and methods

### Animals

p66-knockout (p66-KO) mice (kindly provided by Dr. Marco Giorgio) were maintained as homozygotes in a C57BL/6 background (Migliaccio et al., 1999). Isogenic WT C57BL/6 mice were obtained from The Jackson Laboratory (Bar Harbor, ME). All experimental procedures were conducted following NIH guidelines under an Institutional Animal Care and Use Committee-approved protocol from the Oregon Health and Science University.

### Preparation of post-natal hippocampal neuronal cultures

Dissected hippocampi of p66-KO and WT post-natal mouse pups (P0–P2) were incubated for 30–35 min at 37°C in a solution of 2 mg/mL papain (Worthington Biochemical Corp., Lakewood, NJ) in B27/Neurobasal A medium (Invitrogen, Carlsbad, CA) with 0.5 mM glutamine (Sigma, St. Louis, MO, USA). The hippocampi were then transferred into 2 mL of culture medium [B27/Neurobasal A medium with 0.5 mM glutamine and 50 mg/L gentamicin (Sigma, St. Louis, MO)] and triturated 10 times with a 1000 ul pipette tip, followed by 10 times with a 200 ul pipette tip. Approximately 2 mL of the supernatant was transferred into a new tube, and the remaining tissue was resuspended in another 2 mL of medium and triturated as described above. The cells were counted by hemocytometer and seeded on six well plates pre-coated with 10 μg/mL poly-d-lysine (PDL) (Sigma, St. Louis, MO) at a density of 100,000 cells/well. After 24 h incubation in a humidified incubator at 37°C and 5% CO_2_, the medium was replaced with fresh culture medium.

### Cell viability studies

The hippocampal neuronal cultures were maintained for one week before experimental manipulation. Prior to treatment with either diethylenetriamine/nitric oxide adduct (DETA-NO) or H_2_O_2_ (Sigma, St. Louis, MO), the cells were washed with Neurobasal A medium/0.5 mM glutamine. Half of the six wells were designated control wells and incubated with Neurobasal A medium/0.5 mM glutamine. The other wells were designated treatment wells and incubated with different concentrations of DETA-NO (100, 250, and 500 μM) or H_2_O_2_ (100, 250, 500 μM, and 1 mM) in Neurobasal A medium/0.5 mM glutamine. The DETA-NO treatments were incubated for 1 h at room temperature to allow for the generation and release of nitric oxide. Cells treated with H_2_O_2_ were incubated for 15 min at 37°C, and cells treated with DETA-NO were incubated for 3 h at 37°C. Following the indicated treatment period, the cells were washed twice in Neurobasal A medium/0.5 mM glutamine and returned to the incubator in fresh culture medium. Neuronal viability was assessed 24 h later by incubating the cells in 1 μM Calcein AM (AnaSpec, San Jose, CA) for 30 min at 37°C, and manually counting live neurons based on morphologic appearance and presence of green fluorescent dye at 20X with a fluorescence inverted microscope. Each well was divided and marked into eight regions, and three random areas were counted per region. A total of 72 random areas were counted per treatment or control group per plate. The cell viability percentage was calculated per plate as follows: (live cell count in treatment group)/(live cell count in control group) × 100. All analyses were done blinded to genotype.

Qualitative images of the cell viability studies were taken with either a widefield-inverted microscope at 10X or with a Zeiss LSM710 confocal microscope at 5X.

### Mitochondrial morphology experiments

Post-natal neurons were dissociated from hippocampal tissue as described above, and 1–2 million cells were pelleted and resuspended in 100 μL of nucleofection solution with 3 μg each of a plasmid that directs the expression of GFP within the mitochondrial matrix (Pinton et al., 2007) and a plasmid that directs the expression of mCherry in actin as a neuronal filler (mCherry–β-actin plasmid) (both plasmids generously provided by Dr. Gary Banker at OHSU). The cell/plasmid solution was electroporated following the Amaxa electroporation system protocol (Amaxa, Lonza, Basel, Switzerland) for post-natal neurons using program O-05. Following electroporation, the cells were counted and plated in culture medium at 100,000 cells/well in six well plates, with each well containing a 25 mm glass coverslip coated overnight with 10 μg/mL PDL. After 24 h incubation in a humidified incubator at 37°C and 5% CO_2_, the medium was replaced with fresh culture medium.

Week-old cultures were treated with either the control medium (Neurobasal A medium/0.5 mM glutamine), 25 μM H_2_O_2_, or 500 μM DETA-NO for 1 h at 37°C. The cells were then washed twice with Neurobasal A medium/0.5 mM glutamine and fixed with 4% paraformaldehyde for 20 min at 37°C. Following fixation, the cells were washed twice with 1X PBS, and the coverslips were mounted on slides with Prolong Gold Anti-Fade (Invitrogen, Carlsbad, CA). The slides were imaged with a Zeiss LSM710 confocal microscope using a 63X oil objective. For each randomly chosen neuron, a Z-stack of the axon was imaged (0.38 μm sections; 8–10 sections per neurite). Each z-stack was converted into a 3D image and analyzed by Bitplane Imaris™ software (BitPlane Inc., Saint Paul, MN). To quantify mitochondrial morphology changes, mitochondria in each image were selected by thresholding and analyzed using the Ellipsoid Axis C parameter. Mitochondrial length was defined as the Ellipsoid Axis C parameter × 2. All analyses were done blinded to genotype.

### Mitochondrial ROS production experiments

Post-natal hippocampal neurons were electroporated with mitoGFP, and then plated on 25 mm PDL-coated glass coverslips in six well plates as described above. Week old cells were treated with either the control medium (Neurobasal A medium/0.5 mM glutamine), 25 μM H_2_O_2_, or 500 μM DETA-NO for 1 h at 37°C. Afterwards, the cells were washed twice with Neurobasal A medium/0.5 mM glutamine and incubated with 1 μM Mitosox Red, a fluorescent reporter that monitors mitochondrial superoxide levels (Invitrogen, Carlsbad, CA) at 37°C for 10 min. The cells were then washed twice with Neurobasal A/0.5 mM glutamine, fixed with 4% paraformaldehyde for 20 min at 37°C, and washed twice with 1X PBS. The coverslips were mounted onto slides with Prolong Gold Anti-Fade.

The slides were imaged with a Zeiss LSM710 confocal microscope using a 63X oil objective. Axons of neurons expressing mito-GFP were imaged in both red and green channels to capture mitochondrial GFP expression and corresponding Mitosox Red staining. Images were analyzed using Metamorph software (Molecular Devices, Sunnyvale, CA) to acquire average intensity measurements of randomly selected mitochondria. Between 20 and 30 axonal mitochondria were analyzed per image.

### Statistics

All statistical comparisons between the p66-KO and WT groups were calculated using the Student's *T*-test for groups with unequal variance. Statistical significance was defined as *p* < 0.05.

## Results

### p66-KO neurons are more resistant to oxidative challenges compared to WT neurons

**Figure 1:**
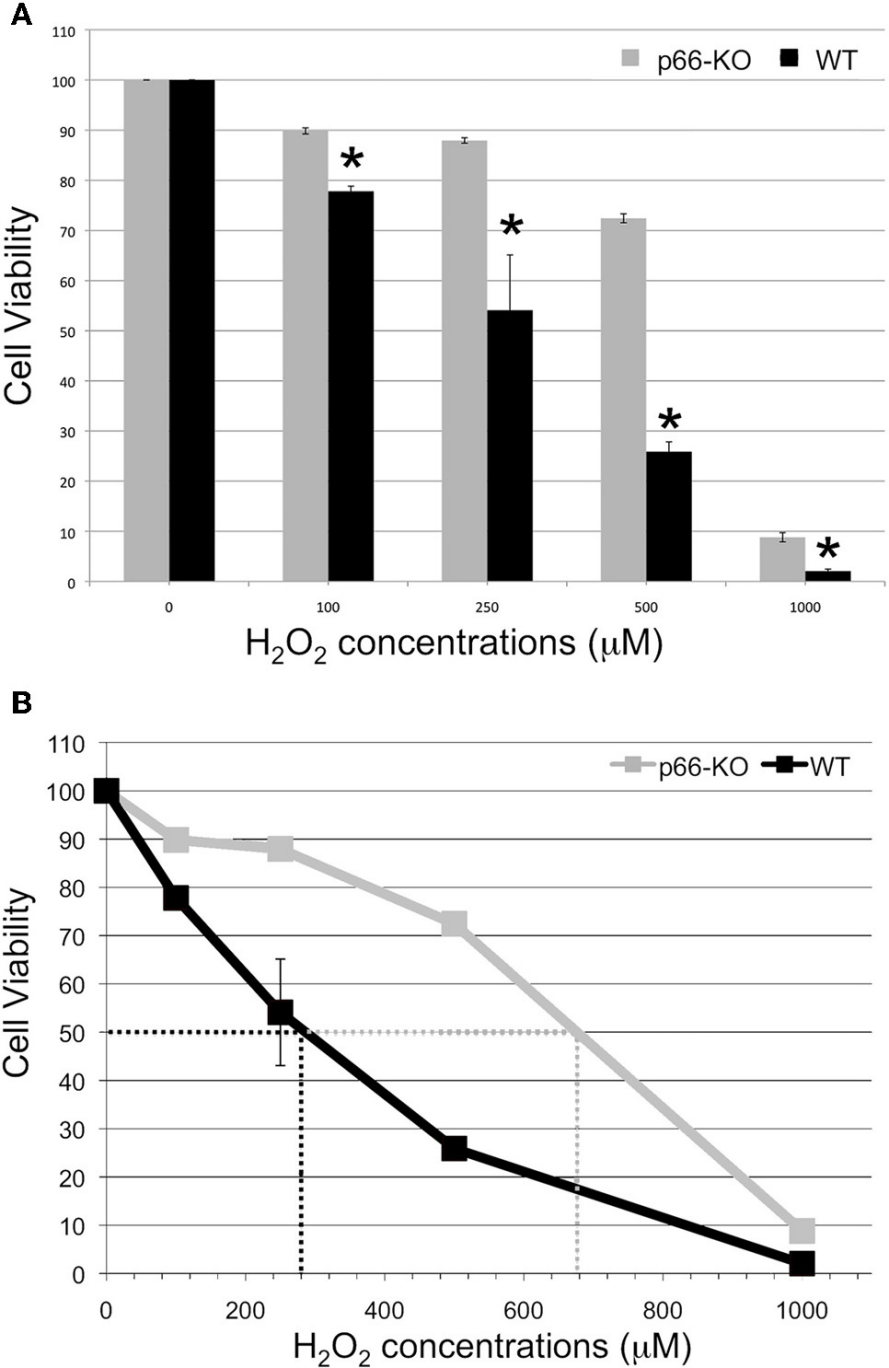
**p66-KO neurons are more resistant to H_2_O_2_ treatment compared to WT neurons.** Week-old p66-KO and WT hippocampal neurons were treated with various concentrations of H_2_O_2_ (100, 250, 500 μM, and 1 mM) for 15 min. Cell counts were obtained 24 h post-treatment using Calcein AM as a viability indicator. **(A)** Cell viability percentages were significantly greater for the p66-KO neurons compared to the WT neurons for the tested H_2_O_2_ concentrations (^*^ = *p* < 0.05). **(B)** H_2_O_2_ concentration vs. survival percentage curves showed that the EC50 for 50% survival was approximately 280 μM for WT neurons and 680 μM for p66-KO neurons, demonstrating that p66 elimination is associated with increased resistance to H_2_O_2_ treatment (*n* per treatment/genotype = 3 cultures; 4 plates/culture).

**Figure 2:**
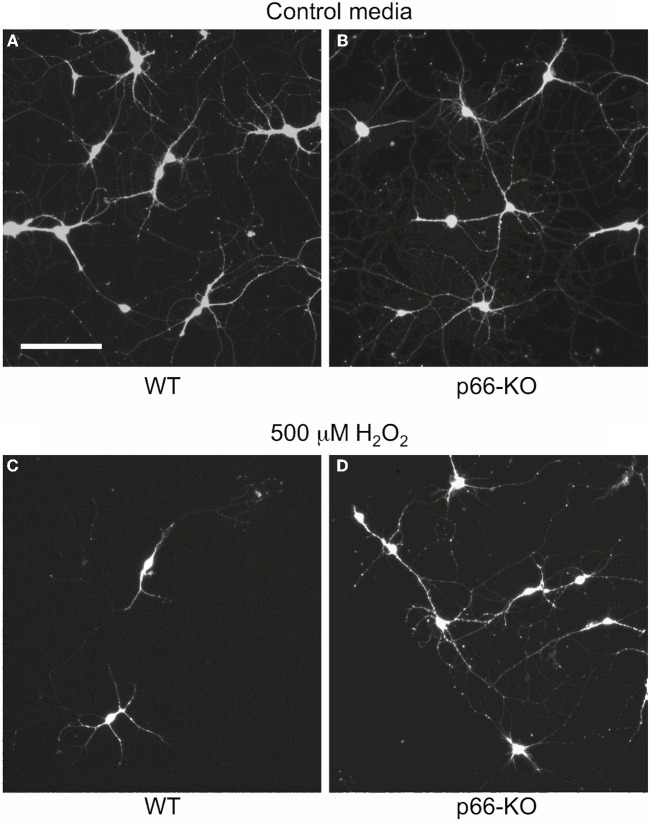
**p66-KO neurons show greater preservation of neuronal structure compared to WT neurons following H_2_O_2_ treatment.** Representative images of week-old p66-KO and WT neurons at 24 h post-treatment with either control media **(A,B)** or 500 μM H_2_O_2_ **(C,D)**; viable cells have been fluorescently labeled with Calcein AM. Images were taken with a wide-field inverted fluorescence microscope using a 10X objective. Note the greater percentage of viable neurons in the p66-KO culture **(D)** compared to the WT culture **(C)** (Bar in *A* = 50 μm).

ROS and RNS species generated by activated microglial cells and immune cells have been proposed to induce mitochondrial dysfunction and neurodegeneration in a variety of neurodegenerative diseases, including MS (Ghafourifar et al., 2008; Su et al., 2009). Therefore, to determine whether p66 elimination protects neurons from the deleterious effects of these stressors, p66-KO, and WT neuronal cultures were treated with physiologic and pathologic levels of H_2_O_2_ and NO, and cell viability was assessed 24 h later (Malinski et al., 1993; Hyslop et al., 1995; Solenski et al., 2003). Elimination of p66 promoted significant neuroprotection in the p66-KO hippocampal cultures following treatment with varying concentrations of either NO or H_2_O_2_. Specifically, a range of physiologic and pathologic H_2_O_2_ concentrations was analyzed (100, 250, 500 μM, and 1 mM), and p66-KO cultures showed significantly greater cell viability compared to WT cultures (Figure 1A). Based on the concentration vs. survival percentage curves generated, the H_2_O_2_-associated EC50 for 50% survival was approximately 280 μM for WT neurons and 680 μM for p66-KO neurons, more than twice the WT concentration (Figure 1B). Representative images of control WT (Figures 2A,C) and p66-KO neurons (Figures 2B,D) further document the preservation of neuronal structure in p66-KO neurons in response to H_2_O_2_ challenges.

**Figure 3:**
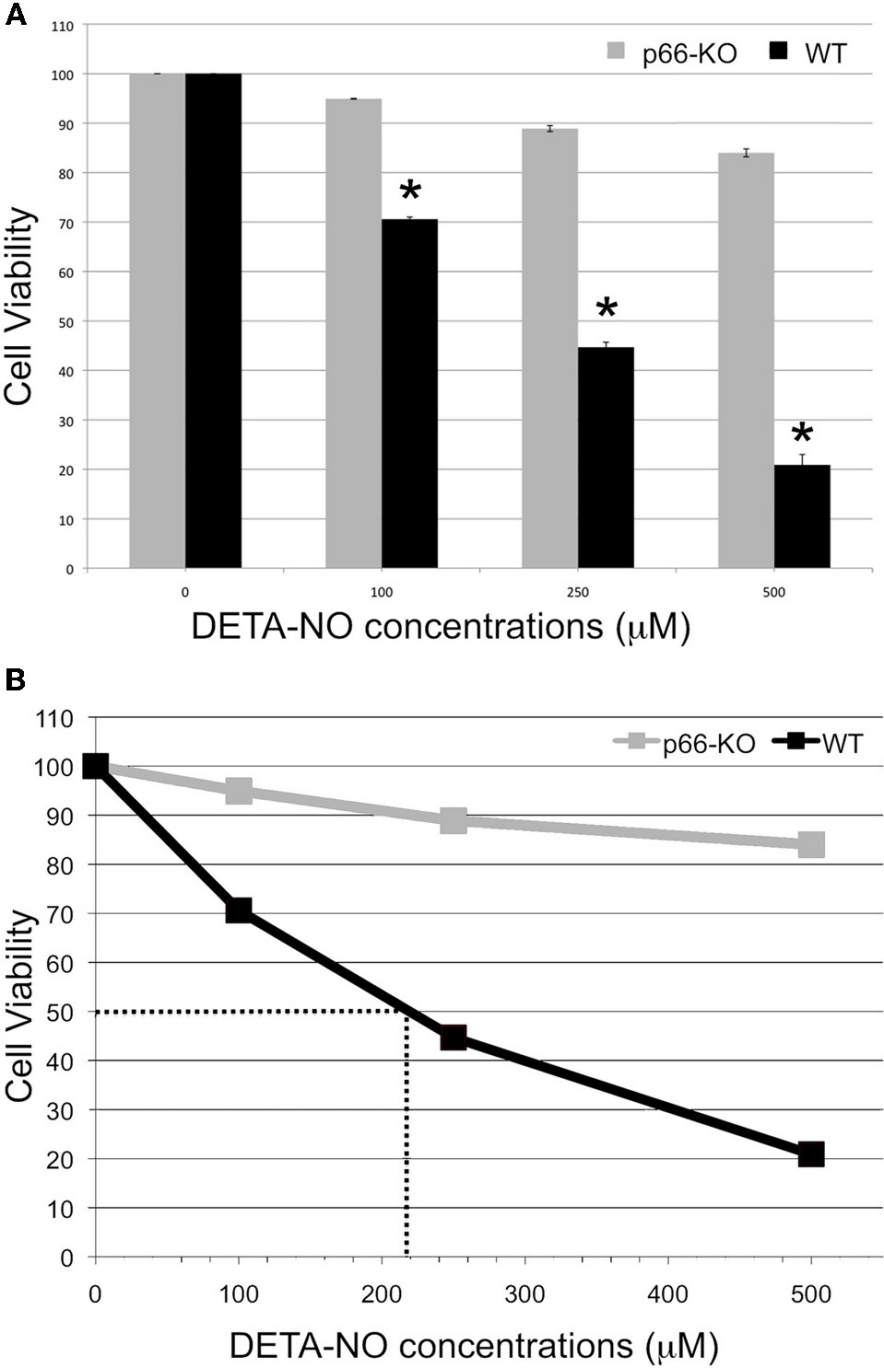
**p66-KO neurons are more resistant to DETA-NO treatment compared to WT neurons.** Week-old p66-KO and WT hippocampal neurons were treated with various concentrations of DETA-NO (100, 250, and 500 μM) for 3 h. Cell counts were obtained 24 h post-treatment using Calcein AM as a viability indicator. **(A)** Cell viability percentages were significantly greater for the p66-KO neurons compared to the WT neurons for the tested DETA-NO concentrations. (^*^ = *p* < 0.05). **(B)** DETA-NO concentration vs. survival percentage curves showed that the EC50 for 50% survival was approximately 220 μM for WT neurons and beyond experimental treatment concentrations for the p66-KO neurons, demonstrating that p66 elimination is associated with considerably increased resistance to DETA-NO treatment (*n* per treatment/genotype = 3 cultures; 4 plates/culture).

**Figure 4:**
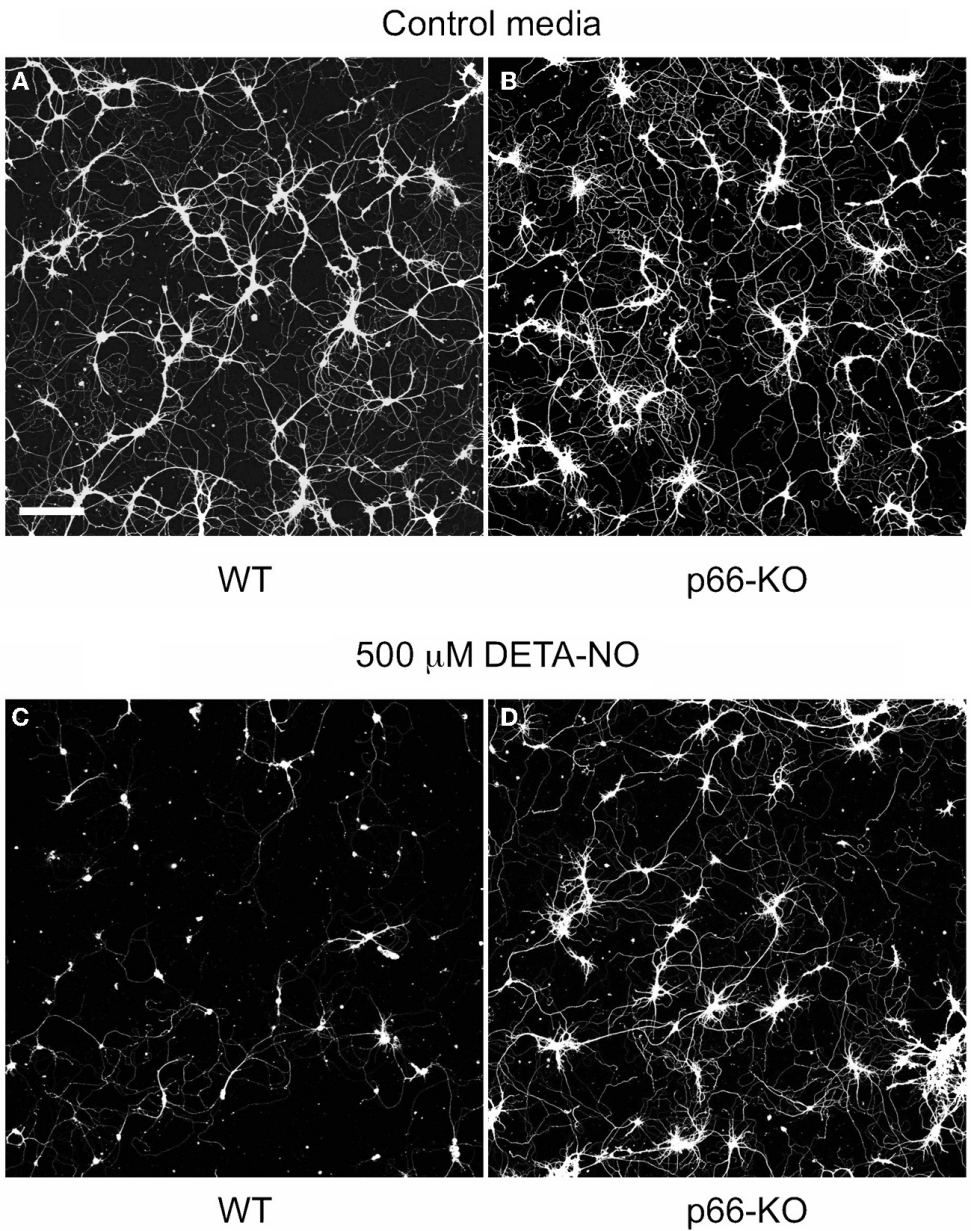
**p66-KO neurons show greater preservation of neuronal structure compared to WT neurons following DETA-NO treatment.** Representative images of week-old p66-KO and WT neurons at 24 h post-treatment with either control media **(A,B)** or 500 μM DETA-NO **(C,D)**; viable cells have been fluorescently labeled with Calcein AM. Images were taken with a laser scanning confocal microscope using a 5X objective. Note the greater percentage of viable neurons in the p66-KO culture **(D)** compared to the WT culture **(C)** (Bar in *A* = 50 μm).

In addition, p66-KO and WT cell cultures were treated with different concentrations of the NO donor DETA-NO. DETA-NO serves as a reliable NO donor since this compound degrades at physiological pH to release NO with predicable first order kinetics (Griffiths et al., 2003). According to previous studies, 1000 μM DETA-NO equates to approximately 7 μM NO after 4 h of incubation, a concentration that is similar to NO levels measured with brain microdialysis during brain ischemia/reperfusion (1–10 μM) (Malinski et al., 1993; Solenski et al., 2003). Therefore, the concentrations of DETA-NO utilized in the cell viability experiments (100–500 μM) likely correspond to levels of NO occurring *in vivo* under physiologic and pathologic conditions. Similar to the H_2_O_2_ results, p66 elimination was found to be associated with significant neuroprotection following DETA-NO treatment (Figure 3A). The DETA-NO-associated EC50 was approximately 220 μM for WT neurons, and beyond experimental treatment concentrations for the p66-KO neurons (Figure 3B). Representative images of control WT (Figures 4A,C) and p66-KO neurons (Figures 4B,D) further document the preservation of neuronal structure in p66-KO neurons in response DETA-NO challenges.

Overall, the viability results indicate that p66 elimination in neurons provides significant protection in response to agents implicated in neurodegenerative pathways, and furthermore, support our previous *in vivo* animal studies (Su et al., 2012).

### p66-KO axonal mitochondria show greater preservation of mitochondrial length following oxidative stress

Mitochondrial morphology has been associated with axonal damage and oxidative stress in both *in vitro* and *in vivo* settings (Pinton et al., 2007; Nikic et al., 2011). In particular, past studies on mouse embryonic fibroblasts (MEFs) treated with H_2_O_2_ demonstrated that mitochondrial morphology was considerably preserved with p66 elimination; mitochondria from p66-KO MEFs treated with H_2_O_2_ remained thin and elongated in structure compared to those of WT MEFs, which were considerably rounder and shortened (Pinton et al., 2007).

**Figure 5:**
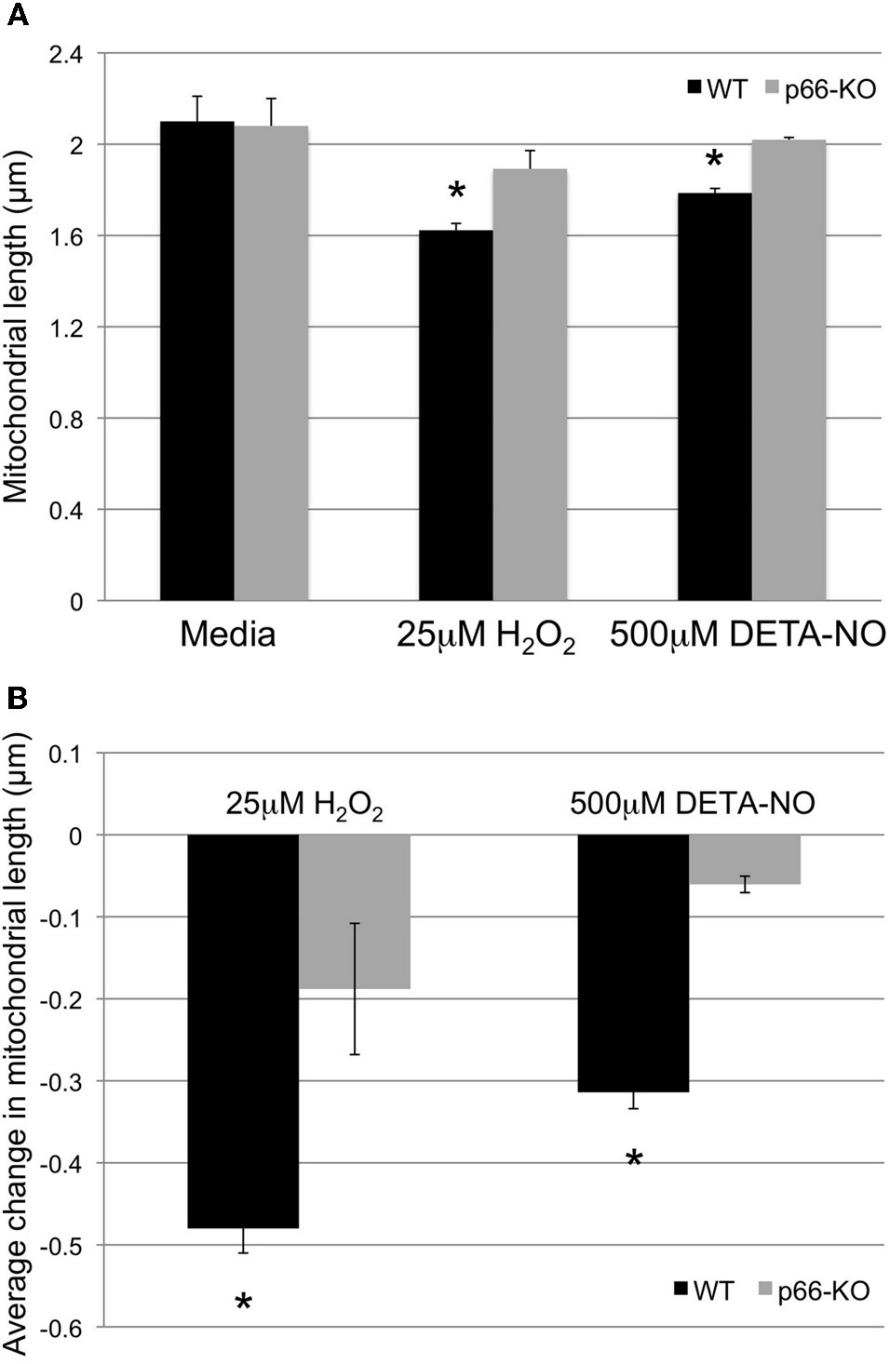
**p66-KO axonal mitochondria show greater preservation of mitochondrial length following oxidative stress.** Mitochondrial morphology changes in week-old p66-KO and WT neurons were compared following treatment with either 25 μM H_2_O_2_, 500 μM DETA-NO, or control media for 1 h. Mitochondria were labeled with mito-GFP for visualization and quantification of morphology changes following treatment. **(A)** p66-KO and WT axonal mitochondria were similar in length following treatment with control media (WT 2.10 ± 0.11 μm, p66-KO 2.08 ± 0.12 μm). Following treatment with H_2_O_2_, axonal mitochondrial length was significantly more preserved in p66-KO neurons (1.89 ± 0.08 μm) compared to WT neurons (1.62 ± 0.03 μm). Similarly, following treatment with DETA-NO, axonal mitochondrial length was significantly more preserved in p66-KO neurons (2.02 ± 0.01 μm) compared to WT neurons (1.79 ± 0.02 μm). **(B)** Quantification of mitochondrial morphology changes following oxidative challenges demonstrated that p66 elimination in neurons was associated with significantly less changes in axonal mitochondrial length (H_2_O_2_ = −0.19 ± 0.08 μm; DETA-NO = −0.06 ± 0.01 μm) compared to WT axonal mitochondria (H_2_O_2_ = −0.48 ± 0.03 μm; DETA-NO = −0.31 ± 0.02 μm). (^*^ = *p* < 0.05) (*n* = 10 images/treatment/genotype).

To assess whether mitochondrial morphology changes in response to H_2_O_2_ and NO challenges are minimized in p66-KO compared to WT neurons, hippocampal cultures were electroporated with mito-GFP and mCherry beta-actin plasmids, and then treated with 25 μM H_2_O_2_, 500 μM DETA-NO, or the control medium for an hour. Neuronal cultures were subsequently analyzed for changes in mitochondrial structure. Utilizing Imaris software, 3D reconstructions of axons were generated from imaged z-stacks, and mitochondrial length within axons was assessed by the ellipsoid axis C parameter (Figure 5). In the control medium-treated neurons, the average mitochondrial length was similar between the WT and p66-KO neurons (WT 2.10 ± 0.11 μm, p66-KO 2.08 ± 0.12 μm; *p* = 0.456). Following treatment with either H_2_O_2_ or DETA-NO, both p66-KO and WT neuronal cultures showed decreases in mitochondrial length, but the decrease was minimal in mitochondria of p66-KO axons. In cultures treated with 25 μM H_2_O_2_, WT mitochondria were shortened by 0.48 ± 0.03 μm compared to 0.19 ± 0.08 μm for the p66-KO mitochondria (*p* = 0.008). The resultant WT mitochondria had an average length of 1.62 ± 0.03 μm compared to 1.89 ± 0.08 μm for the p66-KO mitochondria (*p* = 0.008). Similarly, in cultures treated with 500 μM DETA-NO, WT mitochondria were shortened by 0.31 ± 0.02 μm compared to 0.06 ± 0.01 μm for the p66-KO mitochondria (*p* = 0.0001). Following exposure to DETA-NO, WT mitochondria had an average length of 1.79 ± 0.02 μm compared to 2.02 ± 0.01 μm for the p66-KO mitochondria (*p* = 0.0002). Overall, the results demonstrated that axonal mitochondria were significantly shortened following oxidative challenges of WT neurons compared to p66-KO neurons, suggesting that p66 elimination preserves neurons in part, by the maintenance of mitochondrial integrity under these conditions.

### p66-KO neurons generate less ROS following exposure to oxidative stress

**Figure 6:**
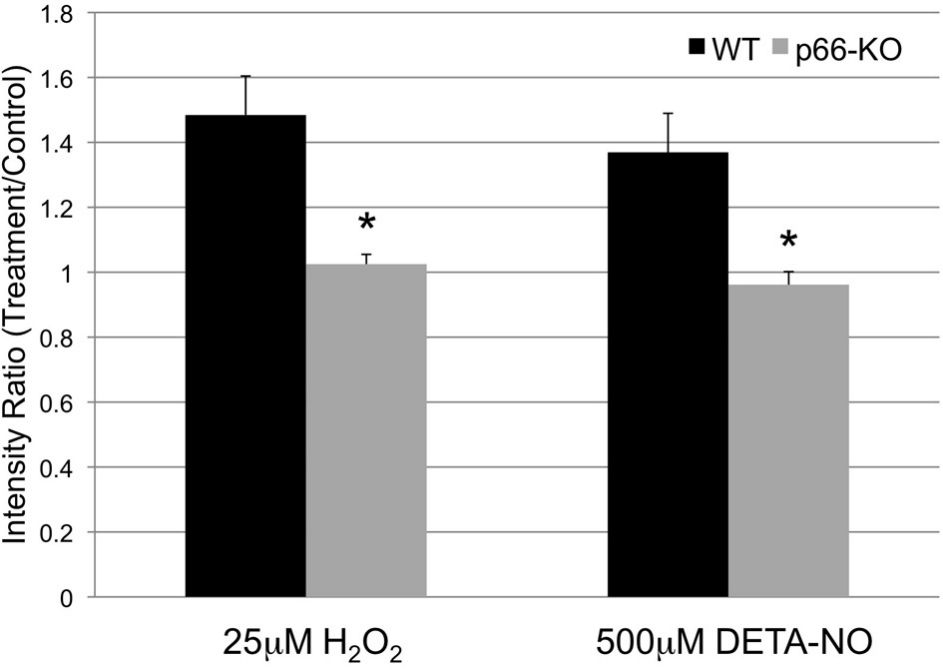
**p66-KO neurons generate less ROS following exposure to oxidative stress.** Week-old p66-KO and WT neurons were treated with either 25 μM H_2_O_2_, 500 μM DETA-NO, or control media for 1 h, and assessed for changes in mitochondrial ROS levels. Mitochondria were visualized with mito-GFP labeling, and mitochondrial ROS was visualized following Mitosox (a fluorescent reporter of mitochondrial superoxide) incubation. Following H_2_O_2_ treatment, p66-KO neurons showed significantly less increases in mitochondrial ROS levels compared to WT neurons (p66-KO = 1.03 ± 0.03X; WT = 1.48 ± 0.12X). Similarly, following DETA-NO treatment, p66-KO neurons showed significantly less increases in mitochondrial ROS levels compared to WT neurons (p66-KO = 0.96 ± 0.04X; WT = 1.37 ± 0.12X). (^*^ = *p* < 0.05) (*n* = 10 images/treatment/genotype).

To determine whether p66 elimination in neurons impacts mitochondrial ROS generation, p66-KO and WT cultures were treated with control medium or individual agents (25 μM H_2_O_2_, 500 μM DETA-NO) and then assessed for changes in ROS levels via Mitosox staining, a fluorescent reporter of mitochondrial superoxide production. The results demonstrated that WT neurons had significantly greater increases in Mitosox intensity levels compared to p66-KO neurons following oxidative challenges (Figure 6). Specifically, WT neurons treated with 25 μM H_2_O_2_ showed a 1.48 ± 0.12X increase in Mitosox intensity compared to a 1.03 ± 0.03X increase in p66-KO neurons (*p* = 0.003). Similarly, WT neurons treated with 500 μM DETA-NO showed a 1.37 ± 0.12X increase in Mitosox intensity compared to minimal change (0.96 ± 0.04X) in p66-KO neurons (*p* = 0.003). Overall, these results suggest that ROS levels increase significantly in WT neurons compared to p66-KO neurons following oxidative insults.

## Discussion

In this report, neurons lacking p66 were demonstrated to be resistant to ROS and RNS stresses implicated in neurodegenerative pathways. Specifically, greater cell viability was demonstrated in p66-KO cultures compared to WT cultures at various treatment concentrations of H_2_O_2_ and DETA-NO, each of which has been demonstrated to drive related apoptotic processes in neurons (e.g., Tamatani et al., 1998; Mailly et al., 1999; Brune, 2005). To further address the underlying mechanisms associated with increased cell viability in the p66-KO neurons, changes in mitochondrial morphology and ROS production were characterized following treatment. The results showed that changes in mitochondrial morphology were less prominent in the p66-KO neurons compared to the WT neurons, with significantly greater preservation of mitochondrial length. In addition, the p66-KO neurons exhibited less elevated mitochondrial ROS levels compared to WT neurons following oxidative insults. Overall, these findings suggest that p66 elimination incurs greater neuronal robustness and preservation of mitochondrial integrity following oxidative insults implicated in neurodegenerative mechanisms.

Importantly, these findings support our current understanding of p66 and its role in cellular responses to oxidative challenges. As outlined earlier, p66 is thought to serve as a ROS sensor and amplifier under conditions of cellular stress by amplifying ROS generation via cytochrome c oxidation and oxygen reduction (Giorgio et al., 2005). Subsequently, elevated ROS has been shown to induce PTP opening as demonstrated by mitochondrial swelling, rupture, and release of cytochrome c to activate apoptotic pathways (Petronilli et al., 1994; Vercesi et al., 1997; Yang et al., 2007). The results reported here are consistent with this proposed pathway, as shown by the higher ROS levels in WT neurons compared to p66-KO neurons following stresses induced by H_2_O_2_ or DETA-NO treatment, demonstrating that p66 elimination reduces ROS amplification following these insults. This, in turn, provides greater preservation of mitochondrial integrity as visualized by mitochondrial morphology. Previous studies have correlated changes in mitochondrial morphology with mitochondrial integrity and activity, including the balance of fission and fusion events and mitophagy of damaged mitochondria. In particular, it has been shown that the dissipation of the mitochondrial membrane potential affects fusion and induces mitochondrial fragmentation (Legros et al., 2002). Furthermore, mitochondrial morphology changes may be correlated to matrix swelling mediated by pathologic PTP opening, release of cytochrome c, and activation of cell death pathways (Scorrano et al., 2002). Assuming that p66 elimination alters the cell crisis signal of elevated mitochondrial ROS, preserves mitochondrial integrity, and subsequently regulates PTP-mediated cell death, this would suggest greater neuronal robustness in p66-KO neurons following these stresses, as is supported by our cell viability studies.

Overall, the characterization of p66 elimination in neurons supports previous studies demonstrating mitochondrial integrity as a key marker of neuronal viability and fate (Nikic et al., 2011). Hence, therapeutics promoting mitochondrial preservation may prove essential to current treatment regimens for neurodegenerative diseases. Mitochondria-targeted drugs are currently being developed, utilizing lipophilic cations or peptides to deliver drug directly to the negatively charged mitochondrial matrix (Sheu et al., 2006; Murphy, 2008). Furthermore, drugs are being developed for specific mitochondrial components such as the PTP via cyclosporin A derivatives targeting the regulatory component CyPD, and ROS via antioxidants such as MitoQ and SS-peptides (Tauskela, 2007; Rocha et al., 2010). The results reported here suggest that pharmacologic inhibition of p66 may also provide neuroprotection by the aforementioned mechanisms, and correspondingly, the results from the *in vivo* studies have demonstrated potential physiologic protection associated with p66 elimination in the context of EAE (Su et al., 2012), MS, and additional neurodegenerative conditions.

### Conflict of interest statement

The authors declare that the research was conducted in the absence of any commercial or financial relationships that could be construed as a potential conflict of interest.
